# Analysis of soil bacterial communities and physicochemical properties associated with Fusarium wilt disease of banana in Malaysia

**DOI:** 10.1038/s41598-022-04886-9

**Published:** 2022-01-19

**Authors:** Fatin Nadiah Jamil, Amalia Mohd Hashim, Mohd Termizi Yusof, Noor Baity Saidi

**Affiliations:** 1 Institute of Biosciences, Universiti Putra Malaysia, 43400 UPM Serdang, Selangor Malaysia; 2 Department of Microbiology, Faculty of Biotechnology and Biomolecular Sciences, Universiti Putra Malaysia, 43400 Serdang, Selangor Malaysia; 3 Halal Products Research Institute, Universiti Putra Malaysia, 43400 UPM Serdang, Selangor Malaysia; 4 Department of Cell and Molecular Biology, Faculty of Biotechnology and Biomolecular Sciences, Universiti Putra Malaysia, 43400 UPM Serdang, Selangor Malaysia

**Keywords:** Plant immunity, Microbiology, Microbial communities

## Abstract

Fusarium wilt (FW) caused by *Fusarium oxysporum* f. sp. *cubense* Tropical Race 4 (TR4) is a soil-borne disease that infects bananas, causing severe economic losses worldwide. To reveal the relationship between bacterial populations and FW, the bacterial communities of healthy and TR4-infected rhizosphere and bulk soils were compared using 16S rRNA gene sequencing. Soil physicochemical properties associated with FW were also analyzed. We found the community structure of bacteria in the healthy and TR4 infected rhizosphere was significantly different compared to bulk soil within the same farm. The rhizosphere soils of infected plants exhibited higher richness and diversity than healthy plant with significant abundance of Proteobacteria. In the healthy rhizosphere soil, beneficial bacteria such as *Burkholderia* and *Streptomyces* spp. were more abundant. Compared to the infected rhizosphere soil, healthy rhizosphere soil was associated with RNA metabolism and transporters pathways and a high level of magnesium and cation exchange capacity. Overall, we reported changes in the key taxa of rhizospheric bacterial communities and soil physicochemical properties of healthy and FW-infected plants, suggesting their potential role as indicators for plant health.

## Introduction

The soil environment is a complex ecosystem that is primarily controlled by soil microbial communities. The composition, diversity, and function of the soil microbial communities are regulated by climate, cultivation methods, soil nutrients, pathogens, and farm management practices^1–6^. They are further shaped by interactions with a host plant's rhizosphere as it offers a niche with increased nutrient availability due to rhizodeposits and intensive microbe-microbe and plant–microbe communication^7,8^. The soil microbial community provides plant communities with many benefits. One of them is suppressing soil-borne diseases by stimulating phytohormones production, competing with soil-borne pathogens for nutrients, direct microbial competition, or activating microbiota-modulated immunity in plants^9,10^. In this case, the rhizosphere is considered the first line of plant defense against soil-borne pathogens. Furthermore, plant species tend to build their defense strategy against soil-borne pathogens through selective stimulation and support of antagonistic microorganisms. Hence, understanding the composition, diversity, function, and network structure of the rhizosphere microbiome in healthy versus infected soils in relation to soil physicochemical such as pH, macro–micronutrient content, and mineral content properties is the important key to controlling the spread of soil-borne disease.

Banana is considered one of the most important fruit crops globally and is produced predominantly in Asia, Latin America, and Africa^11^. Bananas grown for local consumption are generally grown traditionally by smallholder farmers, providing both food and income. Locally planted bananas are the staple food in many tropical countries and play a major role in food security. Fusarium wilt (FW) of banana caused by *Fusarium oxysporum* f. sp. *cubense (Foc)* Tropical Race 4 (TR4) has been impacting banana production worldwide^12,13^. The fungus is difficult to manage due to its long survival potential in the soil, ability to cause disease at low inoculum level, and long incubation period^14^. The latter renders symptomatic plants as the primary parameter for disease progression below and aboveground. Despite the extensive research on FW, banana cultivars resistant to FW are still not widely available at the moment. Alternatively, the use of beneficial microorganisms to control FW in bananas is gaining momentum with increasing reports of promising results in recent years^15–20^.

Different microbiome profiles associated with FW have been reported from studies conducted in the same study area, such as in Hainan, China. Shen et al.^21^ reported a significant enrichment of *Chthonomonas*, *Pseudomonas*, and *Tumebacillus* in FW-suppressive soils. *Bacillus* was identified as the most dominant bacterial group in the disease suppressive soil by^22^ and was mentioned again by^23^ alongside *Lactococcus* and *Pseudomonas*. Tang et al.^24^ showed that crop rotation decreased the incidence of FW and positively shifted the profile of Acidobacteria, Gemmatimonadetes, Firmicutes, and Actinobacteria. In countries with tropical climates like Indonesia, Acidobacteria and Verrucomicrobia phyla were associated with healthy rhizosphere soil^25^. Based on these findings, each study presented a unique soil bacterial composition that necessitated targeted and predictive biocontrol approaches to effectively prevent or control FW in different localities.

In addition to that, the potential for manipulating physical and chemical soil attributes to manage FW has to be taken into account, even though the interactions between soil attributes and disease severity are still not very well understood. The physical structure of soils has been associated with FW in bananas, but comparative studies are scarce, and different results were generated from different sites. In a field survey in Brazil, a positive correlation between clay content and suppression of FW of bananas was identified^26^, while the same soil type is more conducive in India^27^. Among the chemical properties of the soil, pH is generally considered a fundamental variable, and its effects on FW have been shown in several studies^23,26,28^. However, due to its complex interaction with many other soil factors, the reports are often contradictory. Hence, more studies are needed to understand how soil attributes influence FW severity.

In many reported cases of FW infection, symptomatic and asymptomatic bananas have been found in the same crop fields, suggesting a unique interaction between the plant and its microenvironment. We hypothesize that different bacterial community structures and specific soil properties are associated with plant health status. Identifying beneficial bacterial taxa that differentiate healthy and diseased bananas would be the first step to developing disease suppressive soil that utilizes the indigenous soil microbiota to protect bananas against FW infection. To examine the rhizosphere bacterial assembly of symptomatic and asymptomatic bananas compared to bulk soil in this part of the world, a high throughput sequencing of 16S rRNA was adopted. Simultaneously, characteristics of soil physicochemical properties between healthy and FW-infected soils were investigated. Our results offer additional insight into identifying bacterial groups and soil variables associated with FW disease-free soils.

## Results

A section of pseudostem and leaf tissue from banana plants showing typical FW symptoms was collected, and the presence of TR4 was detected using PCR analysis in both pseudostem and leaf tissue samples. Our results showed intact bands at the expected size (463 bp) in the symptomatic plants sampled randomly on the farm. The same band was detected in the positive control lane and was absent in plants with no FW symptoms (Supplementary Fig. 1).

Bacterial communities associated with the rhizosphere and bulk soil of symptomatic and non-symptomatic banana plants were characterized based on the V3-V4 regions of the bacterial 16S rRNA genes. A total of 2.4 million reads were generated following quality filtering and chimeric sequence removal from 20 soil samples, ranging from 117,139 to 291,980 reads for each sample dataset (Supplementary Table 1). The length distribution of trimmed sequences ranged from 262 to 439 bp. All the 20 samples were rarefied to the minimum number of sequences, and they were clustered into 7651 distinct bacterial Operational Taxonomic Units (OTUs), representing a mean Good's coverage of 0.994. The rarefaction curves of all four groups of the sample (infected rhizosphere, RI; infected bulk soil, BI; healthy rhizosphere, RH; and healthy bulk soil, BH) were near saturation, indicating sufficient sequencing depth to cover the bacterial diversity within individual samples (Supplementary Fig. 2a). The infected soils, RI and BI showed a higher number of OTUs than healthy soils, RH and BH (Supplementary Fig. 2b).

**Figure 1:**
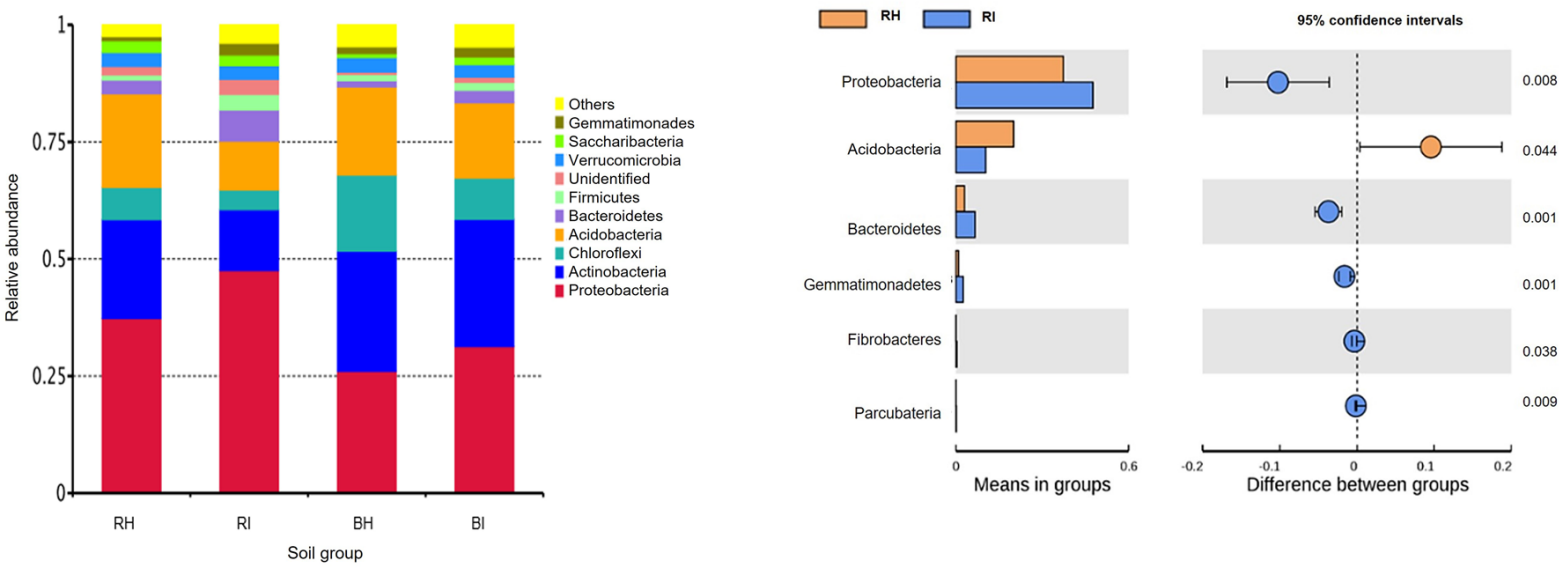
Relative abundance of bacterial phyla associated with individual soil samples. (**a**) A color-coded bar plot shows the percentage of major bacterial phyla. The y-axis represents the classification level of phyla, and the x-axis represents the means value in groups. (**b**) t-test performed at phyla rank for the rhizosphere soil samples. The blue and orange columns represent the average results in the infected and healthy soils, respectively. The colour of the circle agrees with the group whose mean value is higher. The right-most value is the p-value of the significance test between-group variations. Significant differences were shown according to the t-test bar plot taxon rank. BH, bulk soil from healthy plants; BI, bulk soil from infected plants; RH, rhizosphere soil from healthy plant; RI, rhizosphere soil from infected plant.

The bacterial sequences of healthy and infected rhizosphere and bulk soils were assigned at phyla level with Proteobacteria, Actinobacteria, Chloroflexi, Acidobacteria, and Bacteroidetes as major phyla associated with all soils (Fig. 1a). Proteobacteria was the dominant bacterial phylum in all soil samples, representing 26–48% of all bacterial DNA sequences, followed by Actinobacteria (13–27%), Acidobacteria (10–20%), Chlorofexi (4–16%), and Bacteroidetes (1–7%). A few minor phyla ranging from 1–3% (Firmicutes, Verrucomicrobia, Saccharibacteria, Gemmatimonadetes) were also identified, including a small percentage of unassigned sequences. Across the treatments, Proteobacteria phylum was significantly higher in RI at 48% (t-test, *p* < 0.05) (Fig. 1b) compared to RH at 37%. Similarly, in bulk soils, the phylum is higher in BI at 31% than BH at 26%. On the other hand, Acidobacteria was most dominant in RH (20%) and significantly higher than RI (10%) (t-test, *p* < 0.05). The phylum was found in BH at 19%, BI at 16%, and RI at 10%. There was no significant difference between healthy and infected bulk soil for the Proteobacteria and Acidobacteria.

**Figure 2:**
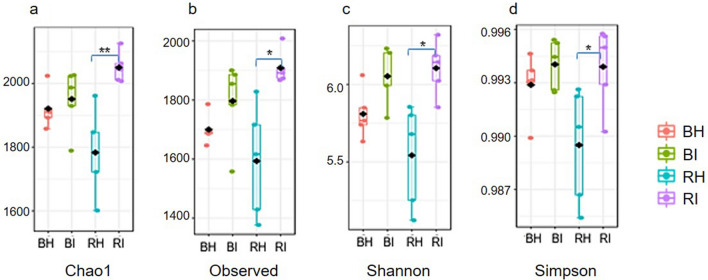
Alpha diversity of the soil bacterial community according to the (**a**) Chao1, (**b**) Observed OTU, (**c**) Shannon and d) Simpson at OTU level represented as boxplot. Each boxplot represents the diversity distribution of a group present within soil type and pairwise comparison was performed using t-test. Significant differences were accepted when *p* < 0.05 between the two groups. * denotes *p* < 0.01 and ** denotes *p* < 0.001.

Bacterial communities were also evaluated using richness and diversity indices (Fig. 2). As a measure of α-diversities, the richness and diversity were consistently higher in the infected soils (RI, BI) than healthy soils (RH, BH) as shown by Chao1, Observed, Shannon and Simpson indices. Significant differences (t-test, *p* < 0.05) were observed in all the indices between RI and RH.

**Figure 3:**
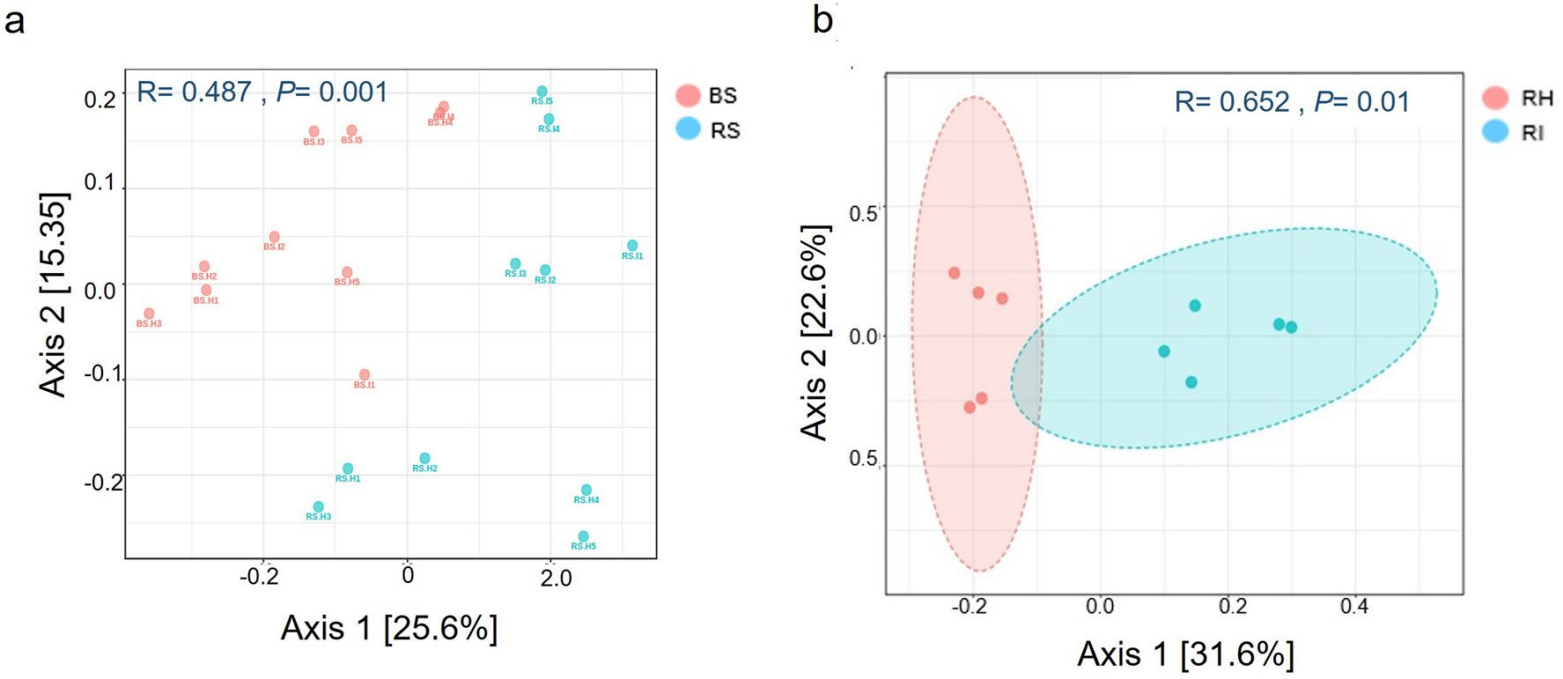
Principal coordinate analysis (PCoA) based on Bray–Curtis distance between (**a**) all soil samples, colored according to soil types (rhizosphere and bulk soils) and (**b**) healthy (RH) and infected (RI) rhizosphere soils.

To measure the changes in species diversity in all soil samples, PCoA was performed based on Bray–Curtis distances on each sample. An analysis of similarity (ANOSIM) testing performed on all sample groups showed dissimilarity between samples (ANOSIM, R = 0.553, *p* < 0.01) (Supplementary Fig. 3a, Supplementary Table 2). Axis 1 and Axis 2 explained 41% of variances among four types of soils (BH, BI, RH, and RI). Samples from rhizosphere and bulk soils exhibited distinct clustering according to soil type (ANOSIM, R = 0.487, *p* < 0.01) (Fig. 3a, Supplementary Table 2). Similarly, samples from rhizosphere soils showed a good separation along the first component, Axis 1, at 31.6%, where RH was clearly distinguished from RI (ANOSIM, R = 0.652, *p* < 0.05) (Fig. 3b, Supplementary Table 2). The community composition for bulk soils, however, was not separated by health status (ANOSIM, R = 0.004, *p* > 0.05) (Supplementary Fig. 3b, Supplementary Table 2).

**Figure 4:**
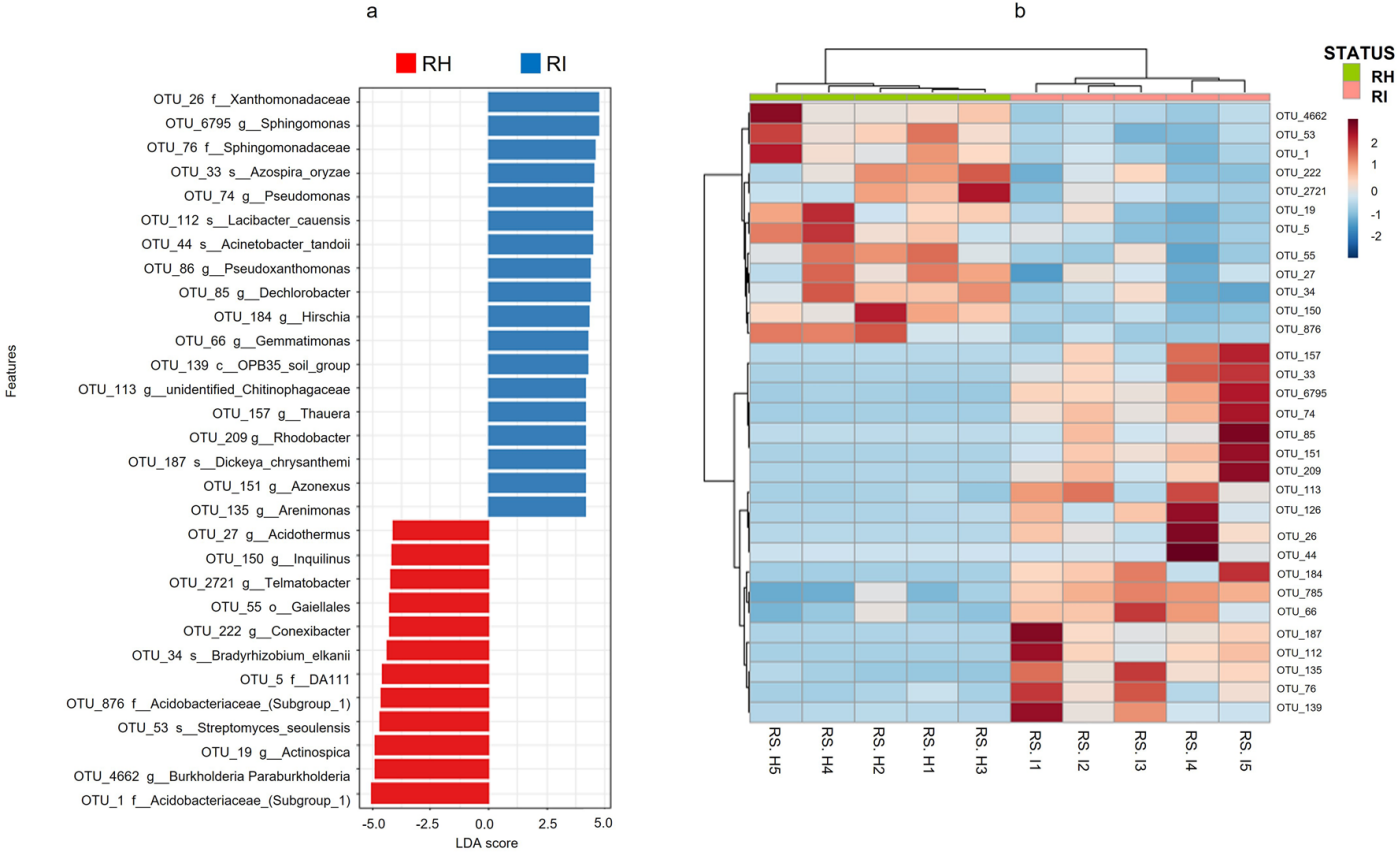
Differential abundance of bacterial taxa in the rhizosphere soil samples as determined by LEfSe. (**a**) Bacterial community between RH and RI at feature-level based on adjusted *p*- value cutoff = 0.05 with LDA score > 4. (**b**) Heatmap and hierarchical cluster analysis of bacterial taxa measured using Euclidean distance and Ward linkage clustering algorithm at feature-level based on the relative abundances of biomarker taxa from RH and RI.

Linear discriminant analysis Effect Size (LEfSe) was used to identify bacterial groups responsible for the differences between RH and RI. LEfSe identified 30 taxa with Linear Discriminant Analysis (LDA) effect size greater than 4 distinguishing RH and RI (Fig. 4a). Out of that, 18 taxa were more abundant in RI, with *Xanthomonadaceae*, *Sphingomonas*, *Azospira oryzae*, *Pseudomonas*, and *Acinetobacter tandoii* as the top discriminating taxa. Meanwhile, the biomarker taxa in RH were *Acidobacteriaceae*, *Burkholderia_paraburkholderia*, *Actinospica*, *Bradyrhizobium elkani*, and *Conexibacter*. Notably, *Burkholderia* and *Streptomyces* were among the highly abundant genera in RH. The heatmap and hierarchical clustering of the biomarker taxa in Fig. 4b revealed a separated cluster of RH and RI, which supported the role of the biomarker taxa in differentiating between the healthy and infected rhizosphere soil samples.

**Figure 5:**
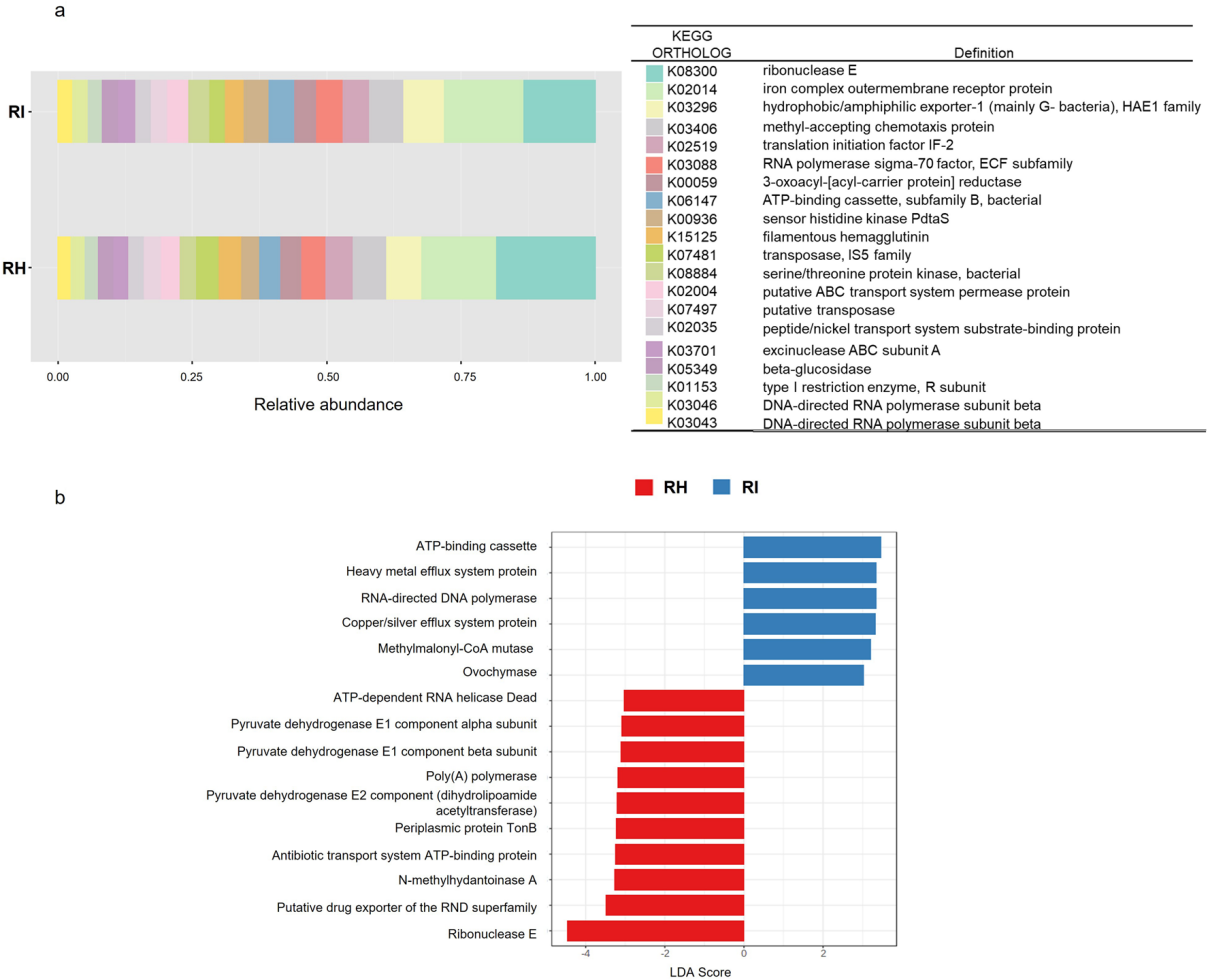
Tax4Fun predictions of the functional composition of rhizosphere microbiome of healthy and FW-infected banana. (**a**) Relative abundances of KEGG functional genes encoded in rhizosphere soils. (**b**) Differentially abundant KEGG functional genes in RH and RI. LDA effect size (LEfSe) was calculated using LDA with *p*-value cutoff = 0.05 with LDA score > 3 of KEGG ortholog.

To evaluate the bacterial functions in the rhizosphere soils, functional gene content was predicted and enumerated using Tax4Fun. A composition of top 20 KEGG functions in rhizosphere soil were shown in Fig. 5a. All the KEGG functions were relatively similar in abundance between RH and RI except for K08300 (ribonuclease E), which was highly abundant in RH (18%) compared to RI at 13%. To identify bacterial functional pathways that may be over or under-represented in healthy and FW infected-soils, supervised comparisons were performed with LEfSe. A total of 16 differentially abundant KEGG orthologs (LDA score > 3) were identified in the rhizosphere soils including K08300 (Fig. 5b). The KEGG functions were clustered according to the health status (Supplementary Fig. 4), indicating their potential as biomarkers to differentiate healthy and infected rhizosphere soil. The results generally confirmed that RH contains more microbiome involved in RNA metabolism and transporters pathways. Meanwhile, heavy metal transport is more enriched in RI. These results demonstrated that changes in soil bacterial community composition induced the alteration of microbial functions in the rhizosphere soil.

In this study, 20 soil physicochemical properties were analyzed. The results showed that there was no significant difference in the physical and chemical properties of healthy and infected soils except for Mg and CEC that were higher in the healthy soil (*p* < 0.05) (Supplementary Table 3). Pearson correlation analysis was undertaken to further explore the relationship between the soil physicochemical variables. Strong positive (> 0.8) and statistically significant correlations (* = *p* < 0.05, ** = *p* < 0.01) were observed between Mg, pH and Fe, C and N, and Mn and Zn in the healthy soils (Supplementary Table 4). Conversely, significant negative correlations were found between Mn and clay. CEC and Mg revealed a strong positive correlation (r = 0.911) in the healthy soils but was not statistically significant. The strongest positive correlation is observed in the infected soils between pH and Ca (r = 0.956**) (Supplementary Table 5). Meanwhile, CEC did not show any significant correlation with other soil properties.

## Discussion

In this study, the alterations of soil bacterial community and the abiotic factors related to FW infection and healthy banana plants were investigated at a plot level. Herman et al.^29^ explained that spatial heterogeneity provides specific niches and creates ecological opportunities. Intensive sampling of local environments increases the chances of detecting rare OTUs due to their low local abundance, habitat specificity, or restricted geographic spread, which can disproportionately influence ecosystem processes. Malard et al.^30^ also highlight the importance of investigating different spatial scales, as drivers at the global scale may not necessarily be the same across the landscape of interest. The soil samples (RH, RI, BH, and BI) from the same banana farm planted with the local susceptible variety, cv. Berangan were analyzed and compared. Since the devastation of banana plantations in 1990 by TR4^31^, the strain has threatened small and commercial banana plantations in Malaysia. In the latest report by^32^, all the 17 isolates of *Foc* collected from nine diseased states in Malaysia were identified as TR4. In our study, the presence of TR4 in all symptomatic plants was confirmed by PCR. However, Berangan bananas are not only associated with vegetative compatibility groups (VCG) of TR4 but also with VCGs of other *Foc* races such as Race 1^33^. Unfortunately, we cannot rule out the absence of other *Foc* races in the infected bananas sampled from the field since no molecular identification was carried out.

We demonstrated that soil bacterial composition and alpha-diversity were different between healthy and FW-infected soils, particularly in the rhizosphere. Interestingly, FW-infected soils (RI and BI) had a greater richness and evenness in bacterial communities, as indicated by observed higher OTU numbers and various alpha-diversity indices with significant differences at the rhizosphere soils (*p* < 0.05) (Supplementary Fig. 2, Fig. 2). Previously^23,34–36^, also reported higher OTU and alpha diversity indices for bacterial communities in the FW-infected soils. Despite the general knowledge that plants may benefit from the diverse microbial communities^37^, we found that healthy soil is not always associated with high bacterial diversity in the soil. Plants can alter their rhizospheric bacterial community via modification of plant exudation patterns in response to pathogen infection, which could explain the higher abundance of bacterial OTU in the infected rhizosphere soil^8,38^. In turn, the bacterial communities can directly influence host plants via various biochemical and physiological activities^38^. Other confounding factors such as the aboveground vegetation and fertilization might influence bacterial community richness independently or synergistically.

The bulk and rhizosphere samples displayed significant separation regardless of health status (ANOSIM, R = 0.487, *p* < 0.001) (Supplementary Figure S3), implying a “rhizosphere effect”. Compared to the bulk zones, plants' rhizosphere zones are rich in nutrients and a hot-spot for microbial communities that may change markedly upon pathogen invasion^39^. The communities are also influenced by numerous other factors such as climate, root exudates, soil types, plant genotype, and developmental stages of a plant. This creates the “rhizosphere effect”, the phenomenon that the rhizosphere microbial community differs from the community in bulk soil due to the recruitment and accumulation of specific microorganisms in the rhizosphere^40,41^. When comparing the bacterial community profiles of healthy and infected soil, the community in the bulk soil did not show apparent segregation (Supplementary Figure S3b). Nevertheless, the composition in the rhizosphere fraction was clustered into distinct groups according to the health status of banana plants (Fig. 3), suggesting that the bacterial community structure in the rhizosphere is markedly altered. This agrees with the diversity (Shannon and Simpson) and richness (Chao1) indices, where the diversity and species richness differ significantly between RH and RI. The correlation between bulk and rhizosphere soil microbiota with FW disease incidence in bananas has also been evaluated^42^. Similarly, it was found that only rhizosphere bacterial community composition correlated with FW disease incidence, accentuating the potential role of rhizosphere bacterial communities in plant defense. Moreover, several studies showed that FW disease suppression in bananas was significantly promoted by applying bio-organic fertilizer that tipped the balance of the rhizospheric microbial community^42–44^.

Considering the significant impact of FW on the community structure of bacteria in the banana rhizosphere, specific bacterial species/genera are expected to become enriched or depleted. Based on Lefse conducted at the feature level, *Acidobacteriaceae* and *Xanthomonadaceae* were identified as the most dominant genera in RH and RI, respectively. OTU belonging to the genus *Acidobacteriaceae* is frequently associated with disease-suppressive soil. They are observed in higher frequencies in the soil suppressive to FW of banana^21,45,46^. *Xanthomonadaceae*, a member of Gammaproteobacteria, has been identified in banana-associated communities in Central America^47^ and present in high abundance in FW-infected soil. The analysis also revealed *Burkholderia* and *Streptomyces* sp. among the significantly enriched genera in RH. The presence of either species has been shown to promote disease suppressiveness even in the presence of pathogens^48–51^. Specific members of *Burkholderia* can act as antifungals due to the production of volatile sulfurous compounds^36^. The volatile compounds were shown to be effective against *F. oxysporum* via degradation of fungal cell walls, cell breakage and leakage of intracellular substances, alterations in hyphal morphology, and ruptured mycelia^52^. The recently isolated *Burkholderia* sp. HQB-1 was suggested as a promising biological agent against the FW of bananas and a plant growth promoter^52^. Likewise, extracts of *Streptomyces* sp. inhibited mycelial growth, spore germination, and hyphae development of TR4^54,55^. Notwithstanding, considering disease suppression is governed by microbial consortia rather than a single taxon, the absence of FW symptoms observed in this study could be partially ascribed to the richness of the *Burkholderia* and *Streptomyces* sp.

As the community structure of bacteria in the banana rhizosphere changes, the predicted gene function of the soil rhizosphere community also altered significantly. RH appeared to differ from RI functionally with respect to RNA degradation and transporters pathways (KEGG level 3). These are essential functions generally required for microorganisms to function and their abundance in RH possibly contributes to the general well-being of the bacterial community to fight off invaders. The abundance of RNA degradation through the activity of ribonucleases suggests that the soil organisms are active^56^. Modulation of mRNA degradation has been associated with various stress conditions in bacteria^57^. Bacterial adaptation to stress involves rapidly regulating transcription, transcript degradation, and translation^58^. Because the goal of an organism is to survive long enough to reproduce, we can assume that in stress conditions—such as pathogen invasion—soil bacteria trigger mechanisms that increase their capability to regulate RNA degradation rapidly. Singh et al.^59^ reported that ribonucleases are involved in phosphate scavenging and recycling and implicated in defense responses to pathogens. Bacteria also evolved membrane adaptation mechanisms in response to the physicochemical change that aids in the cell's survival^60^. In addition to maintaining cellular homeostasis by regulating the intracellular concentrations of ions and solutes, the membrane transport system of bacteria also participates in the secretion of metabolites, including antimicrobial compounds. The active bacterial community of RH may promote health protection to plants by secreting signalling compounds, enzymes, and other interfering metabolites in situ^61^. Notably, heavy metal transport was abundant in RI. The availability and concentration of metals can substantially impact plant-pathogen interactions, where they play important roles in supporting bacterial growth in plant tissues and regulating pathogenesis and virulence genes^62^. Soil rhizobacteria can also alter the chemical properties of the soil, such as pH and organic matter content, to increase metal bioavailability^63^.

The differences in bacterial community composition of healthy and infected soil can be related to changes in soil variables, which can be significantly altered by agricultural practices. In this study, most of the soil variables displayed insignificant differences between healthy and infected soils. Other studies that analyze the association between soil properties and microbial communities in healthy and infected soils have reported a similar observation^64–66^. The soil samples analyzed in this study were collected from a farm that mainly utilizes chemical fertilizers that could lead to less organic matter input. The farm also practiced uniform field management with minimum variation in abiotic environmental conditions, which could explain the insignificant difference between most analyzed physicochemical properties, except for Mg and CEC, which were found significantly higher in healthy soils. Mg is an essential mineral element for plants and microbes and has been associated with FW disease suppression. Going back to 1990, Stover identified magnesium as one of the parameters of healthy soils in banana plantations^67^. The FW pathogens were reported to be less destructive in the presence of adequate Mg by resisting tissue degradation caused by degrading enzymes^68^. In FW of bananas, a higher Mg concentration was associated with a lower average disease incidence and vice versa^69^.

On the other hand, CEC is a measure of soil's ability to hold and exchange cations, including Mg. Therefore, soil with high CEC values is better at retaining essential positively charged nutrients, making them available for the plant. Hence, it is recognized as an important indicator for soil quality^70^. High CEC in healthy soil, as observed in this study, was also reported in other studies. CEC was greater in the healthy soils of several large banana plantations in Indonesia and Australia that practiced integrated pest management (IPM)^71^. Mukhongo et al.^72^ reported that high CEC was conducive to suppressing FW pathogens by *Bacillus* sp. Soil with high CEC values is better at retaining essential positively charged nutrients, making them available for the plant. To boost banana defenses and suppress *Foc* propagules in the soil, Dita et al.^14^ recommended soil pH values that range from 5.6 to 6 and CEC values to be at least 70%. They also insisted that special attention must be paid to Mg content, among others.

## Materials and Methods

### Soil sampling and DNA extraction

Soil samples were collected in April 2018 from a five-acre banana farm in Selangor, Malaysia (3°48′10.1"N 100°50′42.5"E) cultivated with a susceptible variety, cv. Berangan for more than two years. The plants were fertilized with mineral fertilizer. Permission to collect the plant and soil samples was obtained from the Sabak Bernam District Agriculture Office. Experimental research and field studies on the banana plants, including the collection of plant material, complied with the institutional guidelines. Sabak Bernam's main economic activity is agriculture. The plot was previously planted with coconut for several years. Banana plants exhibiting symptoms of FW and non-symptomatic plants in the same farm were selected for sampling. Determination of healthy and infected plants was based on external symptoms (splitting of pseudostem, skirting of wilted leaves and leaf streaking) (Supplementary Fig. 6) and internal symptoms (discoloration of pseudostem and rhizome) (Supplementary Fig. 7) of FW disease. The sampling was conducted in a completely randomized design (CRD) and all the plants (symptomatic and non-symptomatic) were at least 10-15 m away from one another (Supplementary Fig. 5). To detect TR4 in the symptomatic plants, DNA was extracted from pseudostem and leaf tissue using the Wizard Genomic DNA Extraction Kit (Promega, USA). The concentration and quality of the extracted DNA were determined using a spectrophotometer (NanoDrop 2000, Thermo Scientific, USA). The integrity of the DNA was determined by 1% (w/v) agarose gel electrophoresis. PCR was performed using exTEN 2X PCR Mastermix (1st BASE, Malaysia) following the manufacturer's instruction using a specific primer for TR4, FocTR4-F (5’-CACGTTTAAGGTGCCATGAGAG-3’), and FocTR4-R (5’-CGCACGCCAGGACTGCCTCGTGA-3’) by^73^. Samples were prepared in a total volume of 25 mL, containing 50 ng of genomic DNA as a template, 0.2 mM of each forward and reverse primers (FocTR4-F, FocTR4-R), 12.5 mL PCR master mix (1x) (Taq DNA polymerase, dNTPs, MgCl_2_) and 9.5 mL nuclease-free water. The PCR reaction was run in a Peltier Thermal Cycler model PTC-100 with the following program: an initial denaturation of 10 min at 94 °C, followed by 30 cycles of denaturation at 94 °C for 1 min, annealing at 62 °C for 45 s, extension at 72 °C for 45 s and additional extension at 72 °C for 10 min.

Rhizosphere and bulk soil samples were collected from five symptomatic and non-symptomatic plants in the same farm in a completely randomized design. To collect rhizosphere soil, banana roots of 10 cm long (measured from the root tip) were sampled from an individual plant. The roots were shaken by hand to remove any loose soil, leaving only strongly adhered soil, which was considered as the rhizosphere soil. For soil physicochemical analysis, bulk soil at a 0.5 m distance from the individual plant was collected at 20 cm depth using a soil core ring. The root and soil samples were transferred to sterile zip lock bags, kept in an icebox, and brought back to the laboratory for immediate processing. In the laboratory, a sterile blade was used to remove the tightly adhered rhizosphere soil sample and transferred to a microcentrifuge tube for genomic DNA extraction. The bulk soil samples were divided into two parts: one for physicochemical property analysis and one for genomic DNA extraction. For genomic DNA extraction, bulk soil was ground in a sterile mortar and pestle and sieved through a 2-mm sieve before being transferred to a microcentrifuge tube. Total soil DNA was extracted using DNeasy PowerSoil Kit (Qiagen, Germany) following the manufacturer’s protocol. The quantity and quality of the extracted DNA were verified using Qubit 2.0 Fluorometer (Thermo Scientific, USA). The concentration of each DNA sample was > 20 ng/µl, while the purity and quality were in the range of 1.8–2.0 based on A260 /A280 ratio. The DNA integrity was determined by 1% (w/v) TAE agarose gel electrophoresis at 100 V for 60 min. The DNA was stored at − 80 °C before being sent to NovogeneAIT Genomics Singapore PTE LTD (Biopolis, Singapore) for 16S amplicon sequencing.

### 16S rRNA gene amplification using Illumina Hi-seq 2500 PE platform.

The prokaryotic hypervariable V3–V4 region from 16S rRNA gene was amplified using the primers set 341-F (5′ – CCTACGGGNBGCASCAG – 3′) and 805-R (5′- GACTACNVGGGTATCTAATCC- 3′). PCR reactions were carried out with Phusion High-Fidelity PCR Master Mix (New England Biolabs, UK). The same volume of 1 × loading buffer (containing SYB green) was mixed with PCR products, and electrophoresis was operated on 2% agarose gel for detection. PCR products were mixed in equidensity ratios, and a mixture of the PCR products was purified with QIAquick Gel Extraction Kit (Qiagen, Germany). Sequencing libraries were generated using NEBNext Ultra DNA Library Pre Kit for Illumina following the manufacturer's recommendations. The library quality was assessed on the Qubit 2.0 Fluorometer (Thermo Scientific, USA) and sequenced using the Illumina Hi-seq platform, generating 250 bp paired-end reads.

### Sequence processing

Paired-end reads were assigned to samples based on their unique barcode and truncated by cutting off the barcode and primer sequence. Paired-end reads were merged using FLASH V1.2.7 (http://ccb.jhu.edu/software/FLASH/)^74^. Raw tags were analyzed under specific filtering conditions to obtain high-quality clean tags according to the Qiime V1.7.0^75^. The tags were compared with the reference database UCHIME to detect and remove chimera sequences to generate effective tags. Sequence analysis was performed using UPARSE v7.0.1001 (http://drive5.com/uparse/) for all the effective tags^76^. Sequences with ≥ 97% similarity were assigned to the same operational taxonomic unit (OTU), and a representative sequence for each OTU was screened for further annotation. For each representative sequence, MOTHUR software was performed against the SSUrRNA database of SILVA Database (http://www.arb-silva.de/)^77^ for species annotation at each taxonomic rank (kingdom, phylum, class, order, family, genus, species) at threshold (0.8 ~ 1)^78^.

### Bioinformatic analysis and statistical method

Pooled sequences from 5 replicates for each group of soil samples were compared at 97% similarity. A web-based server tool, MicrobiomeAnalyst^79^ was used to analyze alpha and beta diversity, heatmap clustering, differential abundance and functional prediction. Using the Marker Data Profiling tool in the MicrobiomeAnalyst web server, OTU data was initially filtered by default setting (low count filter: min count = 4, prevalence = 20%, low variance filter: 10% inter-quantile range), and scaled using Total Sum Scaling method. The alpha diversity was estimated based on the richness index of Chao1, Observed OTU, Shannon index, and Simpson index. Rarefaction analysis on the obtained OTUs was conducted using MicrobiomeAnalyst to determine the communities' abundance and sequencing data for each sample. Beta diversity between samples was calculated using the Bray–Curtis weighted distance. Principal coordinate analysis (PCoA) using a dissimilarity matrix was applied to visualize the differences between bacterial communities in the healthy and infected soils. Permutational multivariate analysis of variance (PERMANOVA) was used to analyze the data set based on any distance or dissimilarity measures using 999 permutations. In addition to PERMANOVA, analysis of similarities (ANOSIM) was used to give an insight into the degree of separation between the tested groups of samples. Differential abundance analyses of bacteria at different taxa levels between treatments were performed with Linear discriminant analysis Effect Size (LEfSe) and tested using Kruskal–Wallis rank and using Linear Discriminant Analysis (LDA) as implemented in LEfSe^80^. Heatmap of biomarker taxa was constructed based on Euclidean distance and Ward linkage algorithm using MicrobiomeAnalyst platform. Taxa were deemed significant based on their adjusted p-value cutoff = 0.05, and only taxa with LDA score > 4 were visualized. Alpha and beta diversity figures were plotted using the PhyloSeq packages^81^. The metagenomes were predicted from 16S data by Tax4Fun using the Marker Data Profiling tool in MicrobiomeAnalyst. This method enables the mapping of gene abundance profiles, which was predicted from Tax4Fun. The bacterial OTUs were imported into Tax4Fun, and the functional genes were identified from the Kyoto Encyclopedia of Genes and Genomes (KEGG) database^82^. KO data resulted from the Tax4Fun prediction were then imported to Shotgun Data Profiling tool in MicrobiomeAnalyst, filtered by a modified setting (low count filter: min count = 4, prevalence = 20%, low variance filter: 10% inter-quantile range), and normalized using Total Sum Scaling method^83^. LEfSe and heatmap were generated from the KEGG number of the functional genes using the same web tool. KEGG numbers were deemed significant based on their adjusted *p*-value cutoff = 0.05, and only those with LDA score > 3 were visualized. Heatmap of KEGG functional genes was constructed based on Euclidean distance and Ward linkage. All parameters in physicochemical properties were analyzed using a t-test to compare the mean values of physicochemical properties between infected and healthy soils using the SPSS Statistic 23.0 software (IBM, New York, USA).

### Soil physicochemical properties

Bulk soil samples were first air-dried. For pH measurement and nutrient analysis, soil samples were sent to the Soil Fertility Lab, Department of Land Management, Universiti Putra Malaysia. Briefly, soil pH was quantified with a pH meter following the soil being mixed using water (1:5 w/v) for 30 min. Available phosphorus (P) was determined using Bray 2 method. Copper (Cu), zinc (Zn), ferum (Fe), and Manganese (Mn) were determined using the dilute double acid method. Meanwhile, potassium (K), calcium (Ca), and Magnesium (Mg) were determined using ammonium acetate extraction. For mechanical analysis (slit, slay, coarse, fine sand), determination of organic matter (OM), and cation exchange capacity (CEC), the soil samples were sent to MARDILab, Malaysian Agricultural Research, and Development Institute (MARDI). Briefly, CEC was determined using the ammonium acetate method, whereas OM was measured using dry combustion.

## Conclusions

This study demonstrated that the bacterial community composition and diversity differ between healthy and FW-infected soil, especially in the rhizosphere zone. A higher abundance of *Sphingomonas* and *Pseudomonas* was observed in the diseased soils, whereas the *Acidobacteriaceae*, *Burkholderia_paraburkholderia*, *Actinospica*, *Bradyrhizobium elkani*, and *Conexibacter* were enriched in the healthy soils. Notably, *Burkholderia* and *Streptomyces* were among the highly abundant genera in RH. In the soils examined, the health status of the soils is associated with the level of Mg and CEC. Comparisons of bacterial communities and soil physicochemical properties from banana FW diseased and healthy soils will prove essential for constructing disease suppressive soil in the future. However, further research is needed to validate the potential association of Mg and CEC level with FW development and the identified biomarker taxa.

## Supplementary Information

Supplementary Figure S1.

Supplementary Figure S2.

Supplementary Figure S3.

Supplementary Figure S4.

Supplementary Figure S5.

Supplementary Figure S6.

Supplementary Figure S7.

Supplementary Information.

Supplementary Table 1.

Supplementary Table 2.

Supplementary Table 3.

Supplementary Table 4.

Supplementary Table 5.

## Data Availability

The datasets generated and analysed during the current study are available in the National Center for Biotechnology Information (NCBI) repository, https://www.ncbi.nlm.nih.gov/bioproject/725994.
