# Supplementary figures and images for: Analysis of soil bacterial communities and physicochemical properties associated with Fusarium wilt disease of banana in Malaysia
Source: Sci Rep. 2022 Jan 19;12:999. doi: 10.1038/s41598-022-04886-9 (PMC8770495; doi:10.1038/s41598-022-04886-9)

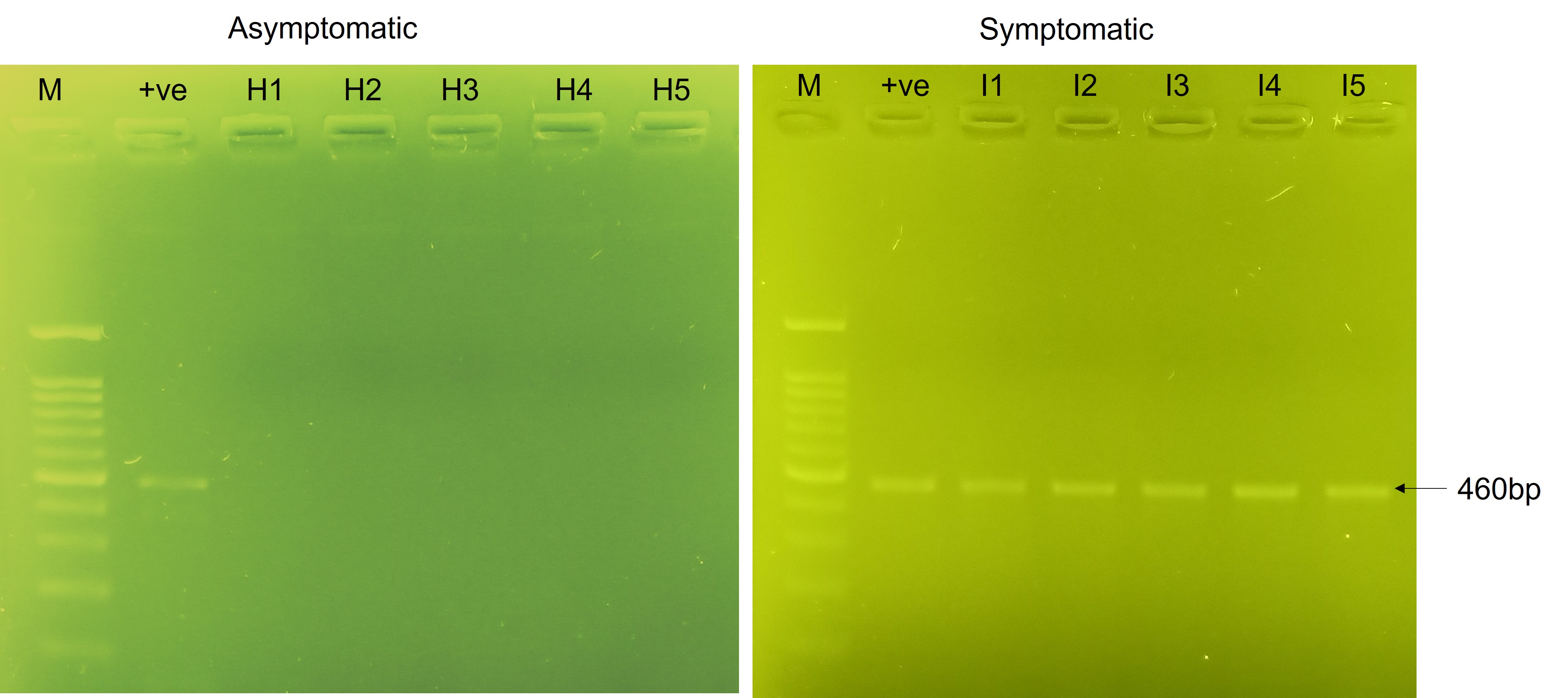

Supplement: Supplementary file 1 — Supplementary Figure S1. [file 41598_2022_4886_MOESM1_ESM.jpg]

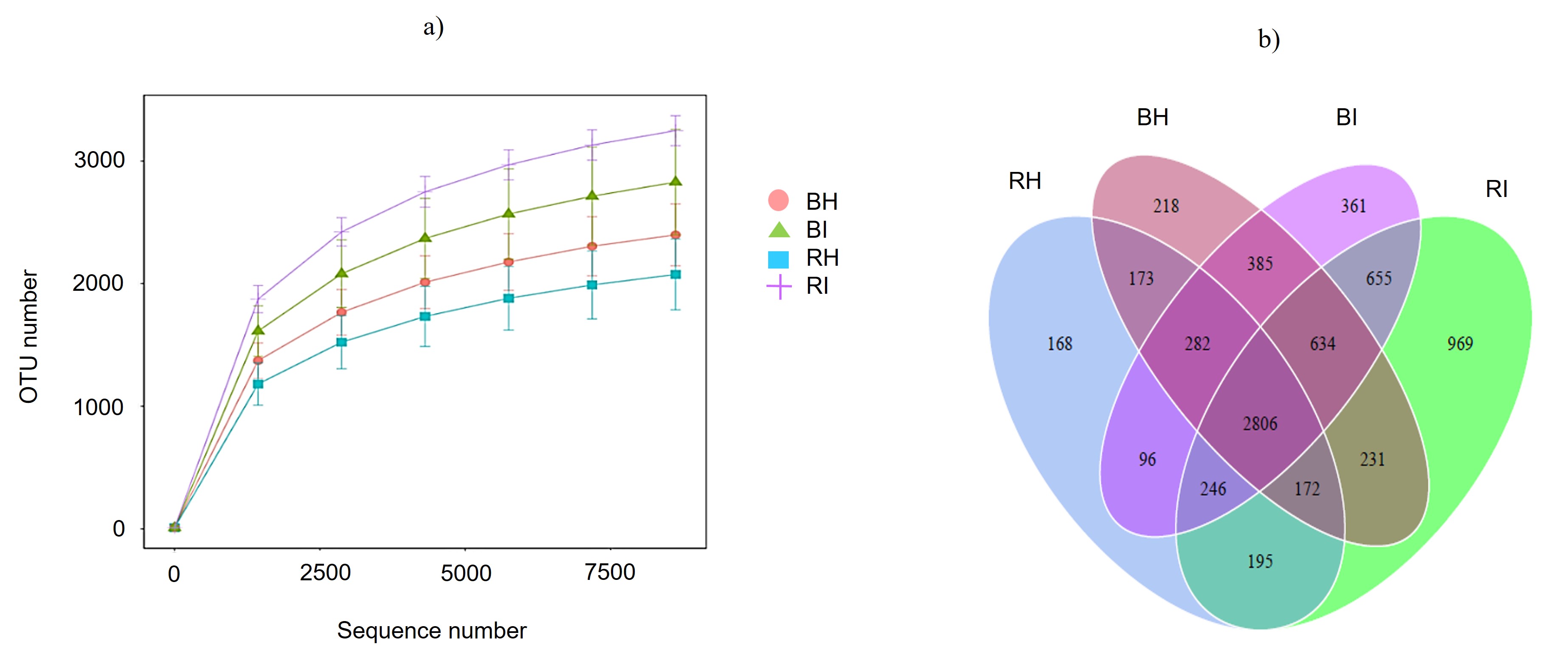

Supplement: Supplementary file 2 — Supplementary Figure S2. [file 41598_2022_4886_MOESM2_ESM.jpg]

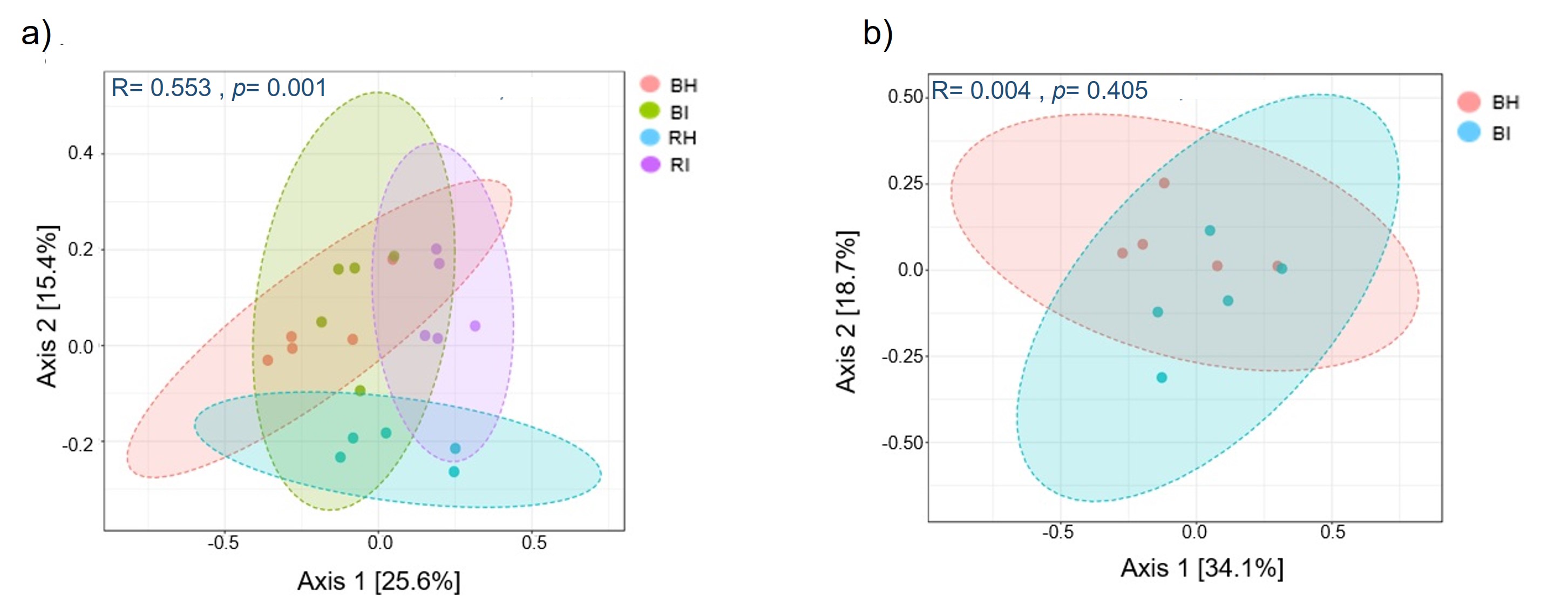

Supplement: Supplementary file 3 — Supplementary Figure S3. [file 41598_2022_4886_MOESM3_ESM.jpg]

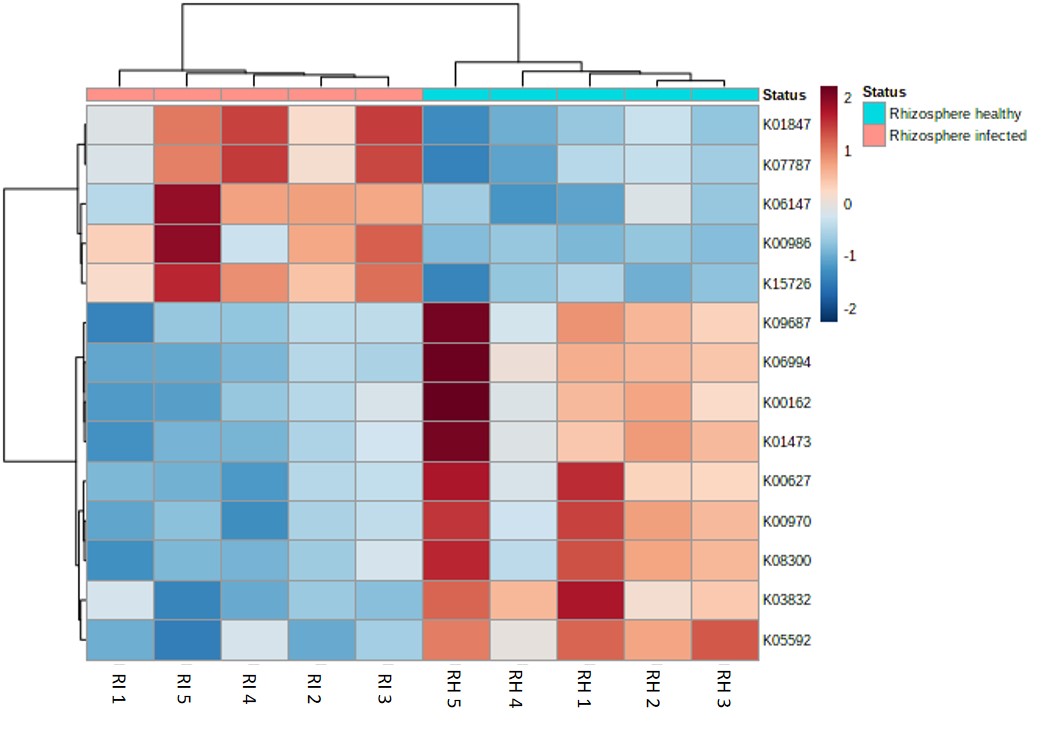

Supplement: Supplementary file 4 — Supplementary Figure S4. [file 41598_2022_4886_MOESM4_ESM.jpg]

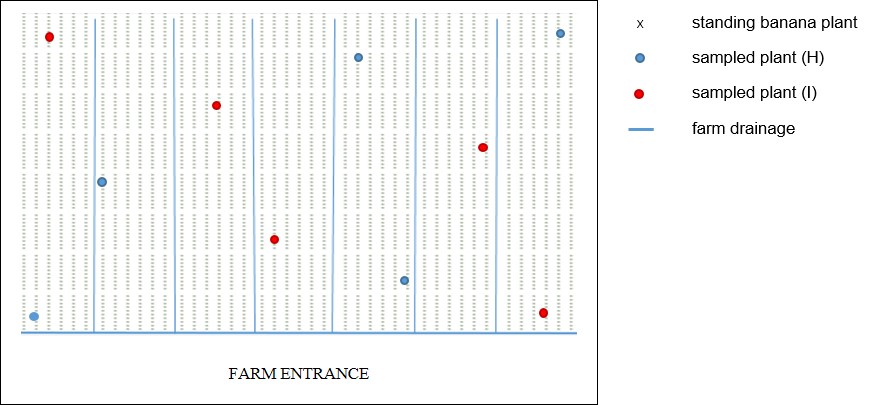

Supplement: Supplementary file 5 — Supplementary Figure S5. [file 41598_2022_4886_MOESM5_ESM.jpg]

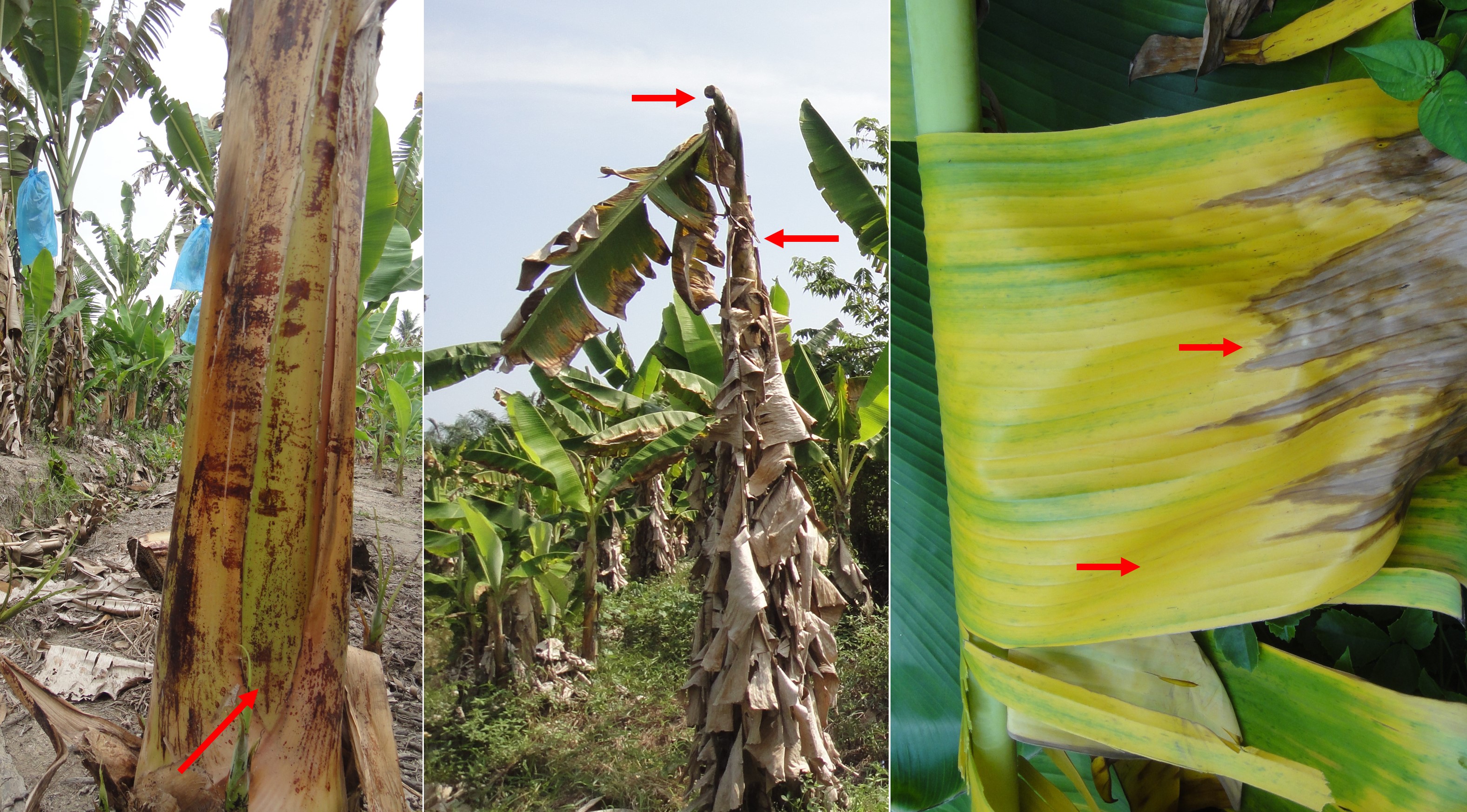

Supplement: Supplementary file 6 — Supplementary Figure S6. [file 41598_2022_4886_MOESM6_ESM.jpg]

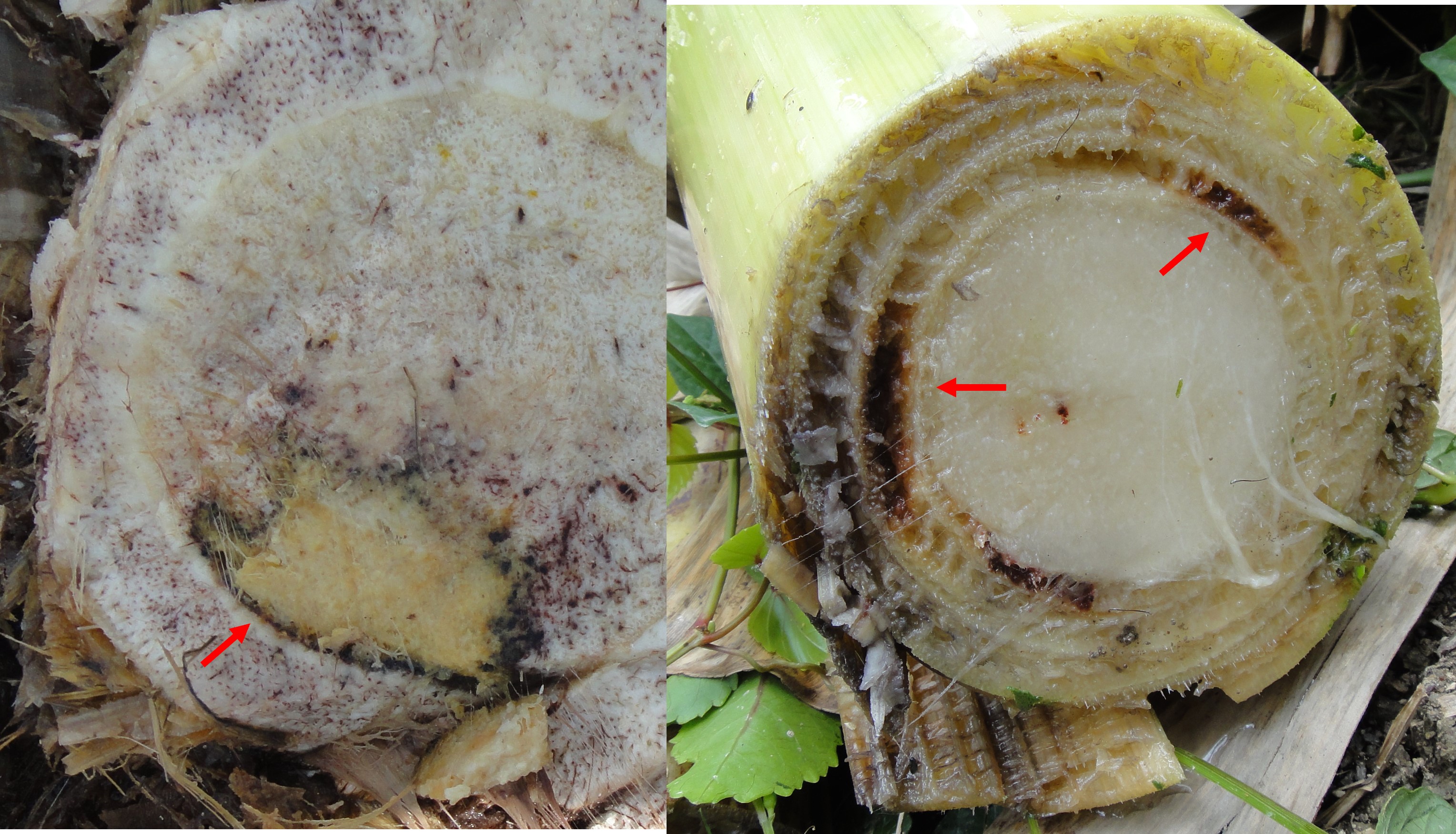

Supplement: Supplementary file 7 — Supplementary Figure S7. [file 41598_2022_4886_MOESM7_ESM.jpg]
