# Supplementary material for: Analysis of soil bacterial communities and physicochemical properties associated with Fusarium wilt disease of banana in Malaysia
Source: Sci Rep. 2022 Jan 19;12:999. doi: 10.1038/s41598-022-04886-9 (PMC8770495; doi:10.1038/s41598-022-04886-9)
Supplement: Supplementary file 8 — Supplementary Information. [file 41598_2022_4886_MOESM8_ESM.docx]

**Supplementary figure legends**

Supplementary Figure 1. Detection of TR4 in the leaves of asymptomatic and symptomatic plants via PCR. Amplification products of PCR using FocTR4R/F with an expected size of 463bp were shown for the positive control and symptomatic plants. No bands were observed in the asymptomatic plants. H1–H5, asymptomatic samples; I1–I5, symptomatic samples; M, 100 bp ladder; +ve, DNA from a pure culture of TR4.

Supplementary Figure 2. Bacterial community profile of healthy and infected rhizosphere and bulk soil of banana plants. a) Rarefaction curves of individual soil samples from a single banana farm. Bacterial OTUs were clustered at 97% similarity level. b) Venn diagram based on OTU. Each sample had 5 replicates (N = 20). BH, bulk soil from healthy plants; BI, bulk soil from infected plants; RH, rhizosphere soil from healthy plant; RI, rhizosphere soil from

infected plant.

Supplementary Figure 3. Principal coordinate analysis (PCoA) based on Bray-Curtis distance between a) all soil samples, colored based on healthy and infected rhizosphere (RH, RI) and bulk soils (BH, BI), and b) healthy (BH) and infected (BI) bulk soils.

Supplementary Figure 4: Heatmap and hierarchical cluster analysis of KEGG functional prediction for rhizosphere soil. The heatmap was constructed in MicrobiomeAnalyst using Euclidean distance and clustered by Ward linkage algorithm. Independent soil groups from RH and RI were shown. The KEGG orthologs were selected based on LEfSe with LDA score > 3.

Supplementary Figure 5. Schematic diagram of a sampling area. A total of ten plants including symptomatic (I) and asymptomatic (H) were sampled. Red dot indicates the location of the sampled plant and soil.

Supplementary Figure 6. External symptoms of FW in cv. Berangan. (a) Split pseudostem. (b) Skirting of wilted leaves. (c) Leaves with yellow and brown streaks. Red arrows indicate external symptoms observed.

Supplementary Figure 7. Internal symptoms of FW in cv. Berangan. (a) Infected rhizome showing golden discoloration. (b) Cross-section of pseudostem showing discoloration. Red arrows indicate discoloration sites.
