# Supplementary material for: Analysis of soil bacterial communities and physicochemical properties associated with Fusarium wilt disease of banana in Malaysia
Source: Sci Rep. 2022 Jan 19;12:999. doi: 10.1038/s41598-022-04886-9 (PMC8770495; doi:10.1038/s41598-022-04886-9)
Supplement: Supplementary file 9 — Supplementary Table 1. [file 41598_2022_4886_MOESM9_ESM.pdf]

Supplementary Table 1. Summary of raw and clean data of 16S sequencing.

Sample name	Total	Merged	Unmerged	Merged (%)	Min length (bp)	Max length (bp)	Average length (bp)
BH 1	158,399	144,548	13,851	91.26	122	441	413
BH 2	156,923	142,073	14,850	90.54	122	441	411
BH 3	153,625	139,347	14,278	90.71	112	441	412
BH 4	150,189	138,050	12,139	91.92	64	441	414
BH 5	140,879	127,601	13,278	90.57	64	441	412
BI 1	117,139	106,390	10,749	90.82	235	441	413
BI 2	117,990	106,901	11,089	90.6	235	441	414
BI 3	159,610	144,238	15,372	90.37	64	441	415
BI 4	146,927	134,751	12,176	91.71	173	441	415
BI 5	138,829	126,046	12,783	90.79	258	441	414
RH 1	140,560	128,226	12,334	91.23	122	441	415
RH 2	142,605	130,012	12,593	91.17	228	441	413
RH 3	291,980	268,245	23,735	91.87	84	443	414
RH 4	144,728	132,903	11,825	91.83	239	442	414
RH 5	145,711	132,963	12,748	91.25	122	439	415
RI 1	145,946	133,172	12,774	91.25	262	441	416
RI 2	155,037	141,054	13,983	90.98	235	441	415
RI 3	141,555	128,936	12,619	91.09	262	440	415
RI 4	159,818	146,046	13,772	91.38	207	441	418
RI 5	151,554	137,277	14,277	90.58	98	441	417
Total	3,060,004	2,788,779	271,225	91.14	262	439	414
