# Supplementary material for: Analysis of soil bacterial communities and physicochemical properties associated with Fusarium wilt disease of banana in Malaysia
Source: Sci Rep. 2022 Jan 19;12:999. doi: 10.1038/s41598-022-04886-9 (PMC8770495; doi:10.1038/s41598-022-04886-9)
Supplement: Supplementary file 10 — Supplementary Table 2. [file 41598_2022_4886_MOESM10_ESM.pdf]

Supplementary Table 2: PERMANOVA table of Principal coordinate analysis (PCoA) based on Bray-Curtis distance between soil groups with different types and health status; Infected (RI) and healthy (RH) rhizosphere soil; Infected (BI) and healthy (BH) bulk soil.

	**F-value**	**R-squared**	**p-value**
All soil (RH, RI, BH, BI)	2.9764	0.35818	0.001**
Rhizosphere and bulk soil (BS, RS)	3.0513	0.2538	0.01*
Rhizosphere soil (RH, RI)	3.3347	0.29421	0.01*
Bulk soil (BH, BI)	0.97396	0.10853	0.422

* denotes $p < 0.01$ and ** denotes $p < 0.001$.
