# Supplementary material for: Analysis of soil bacterial communities and physicochemical properties associated with Fusarium wilt disease of banana in Malaysia
Source: Sci Rep. 2022 Jan 19;12:999. doi: 10.1038/s41598-022-04886-9 (PMC8770495; doi:10.1038/s41598-022-04886-9)
Supplement: Supplementary file 11 — Supplementary Table 3. [file 41598_2022_4886_MOESM11_ESM.pdf]

Supplementary Table 3. Comparison of soil physicochemical properties between healthy and infected soils from banana farm. All data are presented as the mean (standard deviation; $n = 5$).

**Soil properties (unit)**	**Healthy**	**Infected**	***p*-value**
pH	5.087 ± 0.506	5.049 ± 0.435	0.859
MC (%)	6.312 ± 0.574	5.938 ± 0.296	0.084
TOC (%)	1.739 ± 1.064	2.191 ± 0.833	0.123
TON (%)	0.122 ± 0.039	0.137 ± 0.037	0.389
P (mg/kg)	20.832 ± 21.316	20.944 ± 12.103	0.063
K (mg/kg)	213.864 ± 124.779	170.634 ± 34.189	0.853
Ca (mg/kg)	1765.560 ± 267.550	1832.1 ± 390.910	0.662
**Mg** (mg/kg)	**2047.00 ± 414.026**	**1719.5 ± 245.86**	**0.045***
Fe (mg/kg)	477.44 ± 146.104	443.70 ± 99.786	0.554
Mn (mg/kg)	189.86 ± 100.009	166.64 ± 63.428	0.543
Zn (mg/kg)	20.88 ± 4.736	20.92 ± 3.047	0.529
Cu (mg/kg)	0.197 ± 0.112	0.204 ± 0.109	0.889
**CEC** (meq/100g)	**16.241 ± 1.841**	**15.563 ± 0.223**	**0.002****
OM (%)	14.202 ± 1.7017	14.456 ± 0.665	0.218
Clay (%)	63.00 ± 1.581	62.00 ± 4.048	0.476
Coarse Sand (%)	1.00 ± 0.708	0.69 ± 0.360	0.28
Fine sand (%)	19.65 ± 2.399	19.00 ± 1.497	0.477
Silt (%)	28.5 ± 2.934	30.0 ± 3.54	0.19
Bulk density (g/cm3)	0.955 ± 0.042	0.921 ± 0.04	0.075
Air porosity (%)	64.181 ± 1.543	65.255 ± 1.570	0.105

Statistical significance was assessed by T-test and significant differences were accepted when $P < 0.05$ between the two groups. $P < 0.05$ (*) and $P < 0.01$ (**).
