# Supplementary material for: Analysis of soil bacterial communities and physicochemical properties associated with Fusarium wilt disease of banana in Malaysia
Source: Sci Rep. 2022 Jan 19;12:999. doi: 10.1038/s41598-022-04886-9 (PMC8770495; doi:10.1038/s41598-022-04886-9)
Supplement: Supplementary file 12 — Supplementary Table 4. [file 41598_2022_4886_MOESM12_ESM.pdf]

Supplementary Table 4. The Pearson correlation analysis of soil properties in healthy soil.

	PH	MC	C	N	P	K	Ca	Mg	Fe	Mn	Zn	Cu	CEC	OM	Clay	Coarse	Fine	Silt	Bulk density	Air porosity
PH																				
MC	0.186																			
C	-0.39	0.467																		
N	-0.08	0.537	0.880 **																	
P	0.018	-0.029	-0.073	-0.1																
K	0.293	0.671 *	0.704 *	0.750 *	-0.207															
Ca	0.492	-0.154	-0.6	-0.673 *	0.117	-0.277														
Mg	0.876 **	0.268	-0.25	-0.046	0.084	0.35	0.618													
Fe	-0.786 **	-0.178	0.175	0.046	0.002	-0.339	-0.553	-0.810 **												
Mn	0.125	0.383	0.525	0.638 *	-0.094	0.472	-0.158	0.319	-0.479											
Zn	-0.047	0.322	0.404	0.497	-0.094	0.289	-0.23	0.155	-0.333	0.856 **										
Cu	-0.515	-0.594	-0.209	-0.584	-0.205	-0.442	0.207	0.184	0.377	-0.555	-0.525									
CEC	0.219	0.214	0.229	0.191	0.1	0.450	-0.066	0.911	0.079	-0.376	-0.684	0.058								
OM	0.399	0.505	0.262	0.606	-0.317	0.415	-0.246	0.424	-0.318	0.649	0.431	-0.739	-0.02							
Clay	-0.137	-0.124	-0.313	-0.516	-0.173	-0.217	0.312	0.771	0.402	-.0743 *	-0.8	0.666	0.436	-0.5						
Coarse	0.429	0.304	-0.07	0.247	0.218	0.015	0.038	0.333	-0.224	0.376	0.068	-0.625	0.088	0.745	-0.273					
Fine	-0.56	-0.217	-.242	-0.315	0.134	-0.681	-0.093	0.063	0.726	-0.523	-0.22	0.238	-0.258	-0.324	0.256	-0.137				
Silt	-0.104	-0.064	0.242	0.298	0.845	-0.027	-0.246	0.986	0.112	0.219	0.177	-0.352	-0.007	-0.082	-0.419	0.281	0.012			
Bulk density	-0.041	-0.05	-0.443	-0.604	0.329	-0.369	0.475	0.93	0.325	-0.766	-0.691	0.397	0.299	-0.614	0.8	-0.177	0.474	-0.045		
Air porosity	-0.050	0.111	0.355	0.437	-0.408	0.293	-0.442	0.73	-0.284	0.678	0.572	-0.204	-0.274	0.554	-0.598	0.166	-0.492 *	-0.133	-0.907	
