# Supplementary material for: Analysis of soil bacterial communities and physicochemical properties associated with Fusarium wilt disease of banana in Malaysia
Source: Sci Rep. 2022 Jan 19;12:999. doi: 10.1038/s41598-022-04886-9 (PMC8770495; doi:10.1038/s41598-022-04886-9)
Supplement: Supplementary file 13 — Supplementary Table 5. [file 41598_2022_4886_MOESM13_ESM.pdf]

Supplementary Table 5. The Pearson correlation analysis of soil properties in infected soil.

	PH	MC	C	N	P	K	Ca	Mg	Fe	Mn	Zn	Cu	CEC	OM	Clay	Coarse	Fine	Silt	Bulk density	Air porosity
PH																				
MC	0.414																			
C	-0.33	-0.105																		
N	0.02	-0.61	0.468																	
P	-0.730*	-0.335	0.623	0.154																
K	-0.038	-0.396	-.645*	0.052	-0.251															
Ca	0.956**	0.27	-0.254	0.137	-.688*	0.03														
Mg	0.398	0.297	0.253	0.187	-0.42	-0.226	0.384													
Fe	0.32	0.012	-0.466	0.04	-0.476	0.044	0.252	-0.3												
Mn	-0.164	0.253	0.403	0.226	-0.087	-0.095	-0.183	0.646*	-0.312											
Zn	0.713*	0.056	-0.443	-0.103	-0.431	0.109	0.742*	-0.248	0.35	-0.707*										
Cu	0.609	0.3	-0.692	-0.346	-0.494	0.143	0.571	-0.37	0.619	-0.663	0.795*									
CEC	-0.125	0.06	0.131	0.255	-0.048	-0.152	-0.256	0.448	0.193	0.539	-0.671	-0.36								
OM	-0.732	-0.01	0.33	-0.234	0.75	0.01	-0.706	-0.366	-0.421	0.116	-0.563	-0.442	-0.013							
Clay	0.148	0.456	-0.274	-0.233	-0.268	-0.165	-0.131	0.113	0.397	0.239	-0.202	0.118	0.62	-0.178						
Coarse	-0.62	0.135	0.713	-0.069	0.654	-0.553	-0.651*	-0.103	-0.298	0.378	-0.6	-0.621	0.126	0.699*	0.053					
Fine	-0.704	-0.846	0.3	0.484	0.401	0.144	-0.588	-0.215	-0.038	0.077	-0.428	-0.559	0.139	0.158	-0.298	0.237				
Silt	-0.101	-0.09	0.289	0.085	-0.054	-0.074	0.046	0.476	-0.614	0.347	-0.15	-0.38	-0.201	-0.196	-0.485	-0.065	0.228			
Bulk density	0.36	0.624	-0.325	-0.383	-0.29	0.036	0.277	-0.254	0.463	-0.061	0.295	0.531	-0.14	0.14	0.309	0.069	-0.65	-0.642		
Air porosity	-0.369	-0.58	0.346	0.345	0.288	-0.071	-0.29	0.294	-0.491	0.098	-0.329	-0.561	0.156	-0.119	-0.299	-0.032	0.633	0.664	-0.997	
